# Hepatocellular Carcinoma Masquerading as a Large Renal Mass with Hepatic Invasion

**DOI:** 10.1100/tsw.2010.35

**Published:** 2010-02-17

**Authors:** Joseph R. N. Zabell, Kenneth G. Nepple, Neal W. Wilkinson, Laila Dahmoush, Richard D. Williams

**Affiliations:** ^1^ Department of Urology, University of Iowa, Iowa City, IA, USA; ^2^ Department of Surgery, University of Iowa, Iowa City, IA, USA; ^3^ Department of Pathology, University of Iowa, Iowa City, IA, USA

**Keywords:** hepatocellular carcinoma, renal cell carcinoma, renal mass, liver mass, diagnostic imaging

## Abstract

Large masses are evaluated with imaging to assess primary origin and tumor spread. We present the unusual case of a 53-year-old male with a 17-cm right upper quadrant mass suspected to be renal or adrenal in origin based on radiographic findings. After surgical excision, the mass was subsequently discovered to be primary hepatocellular carcinoma with direct extension to the kidney and adrenal gland. A diagnosis of chronic hepatitis B was made postoperatively. Primary hepatocellular carcinoma with direct renal extension is an exceedingly rare occurrence based on our experience and review of the published literature.

## INTRODUCTION

Large renal masses are most frequently primary renal cell carcinoma (RCC). Preoperative imaging with CT, MRI, or ultrasound provides insight regarding the size and anatomy of the primary lesion and possible invasion into adjacent structures. However, imaging does have limitations, as patients thought to have invasion of adjacent organs are often downstaged based on operative findings[1]. Radiographic limitations can lead to incorrect determination of the primary organ of origin for a tumor in the context of metastatic disease. Tumor markers and clinical risk factors may improve on these radiographic limitations. We present the unusual case of a large right upper quadrant mass believed to be renal or adrenal in origin with secondary extension into the liver that was subsequently discovered to be primary hepatocellular carcinoma (HCC) with direct extrahepatic extension into both the kidney and adjacent adrenal.

## CASE PRESENTATION AND MANAGEMENT

A 53-year-old African American male, originally from Ghana, presented for evaluation of a right upper quadrant mass discovered on imaging for back pain. CT (Fig. 1) demonstrated a 13.5- × 9.1- × 17.0-cm mass believed to be a renal vs. adrenal primary tumor with hepatic invasion. To further evaluate the mass, an MRI (Fig. 2) was obtained that could not localize the mass with certainty to the adrenal gland or kidney.

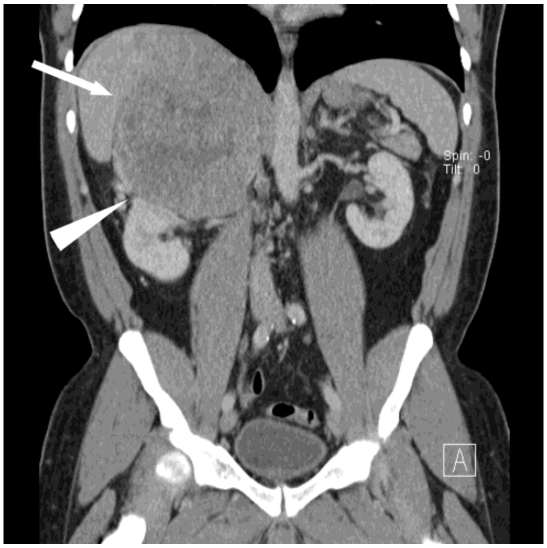

**FIGURE 1.** CT of large right upper quadrant mass. Arrowhead demonstrates tumor and kidney interface. Arrow demonstrates proximity/involvement of right liver.

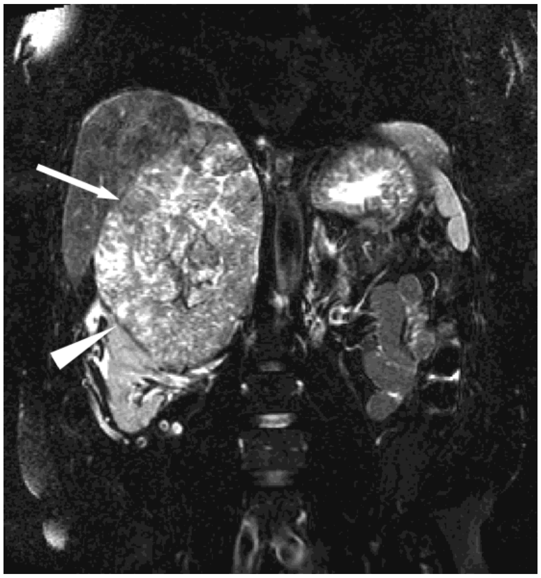

**FIGURE 2.** MRI demonstrating involvement of right kidney and liver. Arrowhead demonstrates tumor and kidney interface. Arrow demonstrates proximity/involvement of right liver.

Direct hepatic invasion was seen with the mass encasing hepatic venous branches, with close proximity to the inferior vena cava (IVC), without involvement of the right portal vein (Fig. 3). Venous tumor thrombus was found within an accessory right renal vein vs. adrenal vein, with slight protrusion of tumor thrombus into the inferior vena cava, but no tumor thrombus was seen in the main renal vein. No radiographic signs of liver cirrhosis or portal hypertension were noted. Metabolic workup demonstrated no evidence of a functional adrenal mass with normal plasma-free metanephrine level, as well as normal urine norepinephrine, epinephrine, metanephrines, and vanillylmandelic acid. Plasma cortisol, serum aldosterone, urine aldosterone, and 17-hydroxyketosteroids were within normal limits. Preoperative liver function tests

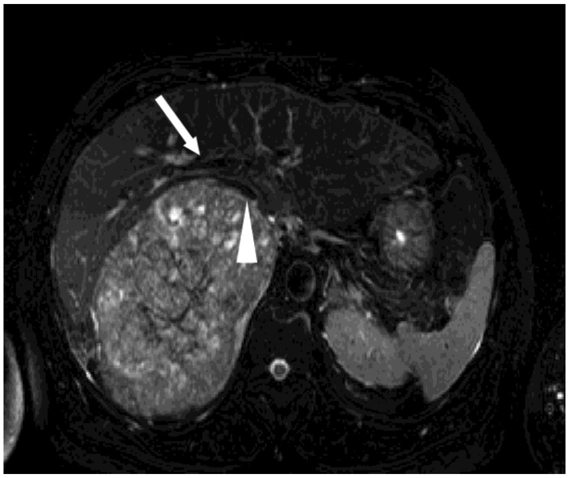

**FIGURE 3.** MRI shows mass in close proximity to IVC. Arrowhead demonstrates tumor and proximity/involvement of vena cava. Arrow demonstrates clear space between right portal vein.

(LFT) showed normal total bilirubin and alanine aminotransferase with mild elevation of alkaline phosphatase 145, gamma-glutamyltranspeptidase 81, and aspartate aminotransferase 68. Chest CT and bone scan were negative for metastatic disease.

A multidisciplinary operative team was mobilized, including urology, surgical oncology, and vascular surgery. Using a thoracoabdominal approach, the patient underwent right radical nephrectomy, including right adrenalectomy, venous thrombectomy, and enbloc resection of the involved diaphragm and the right posterior segments of the liver (segments 6 and 7). The liver demonstrated no signs of cirrhosis. Estimated blood loss from the procedure was 2,500 cc and the patient received four units of packed red blood cells and two units of fresh frozen plasma intraoperatively.

Surgical pathology subsequently demonstrated a 22.5-cm (in greatest dimension) mass consistent with HCC (well to moderately differentiated) with direct extension into the kidney. Microscopic examination showed that tumor cells had abundant granular cytoplasm, round nuclei, and prominent nucleoli, which are features characteristic of hepatocytes (Figs. 4 and 5). Diagnosis of HCC was confirmed by Hepar immunostain positive in tumor cells (Fig. 6). Extensive lymphovascular and portal venous invasion with tumor was present in the liver. All surgical margins, including liver and diaphragm, were negative. Non-neoplastic liver showed chronic portal inflammation and mild to focally moderate fibrosis. A diagnosis of chronic hepatitis B was made postoperatively (positive hepatitis B core antibody and antigen) with low quantitative hepatitis B viral load of 63 IU/ml (1.8 log IU/ml). Hepatitis C serology was negative. The patient had a prolonged hospital course, including intensive care unit stay, acute renal failure from acute tubular necrosis that did not require dialysis, nonocclusive main portal vein thrombosis requiring anticoagulation, and high-output chest tube drainage up to several liters per day from peritoneal pleural communication.

During follow-up at 9 months postoperatively, the patient experienced a rise in alpha-fetoprotein (AFP) from 3.3 ng/ml immediately postoperative to 25 ng/ml (normal <8.9). Chest CT demonstrated bilateral small pulmonary nodules measuring up to 9 mm and a liver MRI showed four new liver densities measuring up to 20 mm, consistent with metastatic disease. Liver metastases were treated with hepatic transarterial infusion chemotherapy using cisplatin/adriamycin with lipiodol, without embolization as no definitive liver lesion was seen on arteriography. The largest liver lesion received external beam radiation therapy of 30 Gy over five fractions. The patient was placed on erlotinib (tyrosine kinase inhibitor) and bevacizumab (vascular endothelial growth factor inhibitor), and was doing well with normalization of his AFP. As of his most recent follow-up at 16 months postoperatively, the patient was continuing with systemic therapy with bevacizumab.

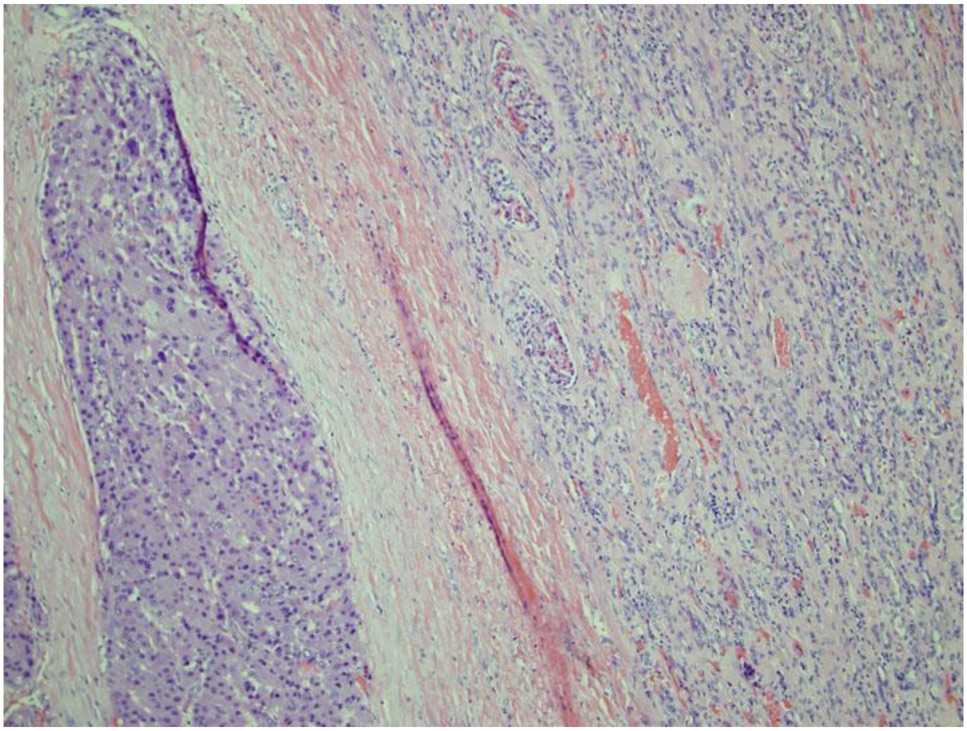

**FIGURE 4.** Histologic section showing renal parenchyma on the right and HCC on the left.

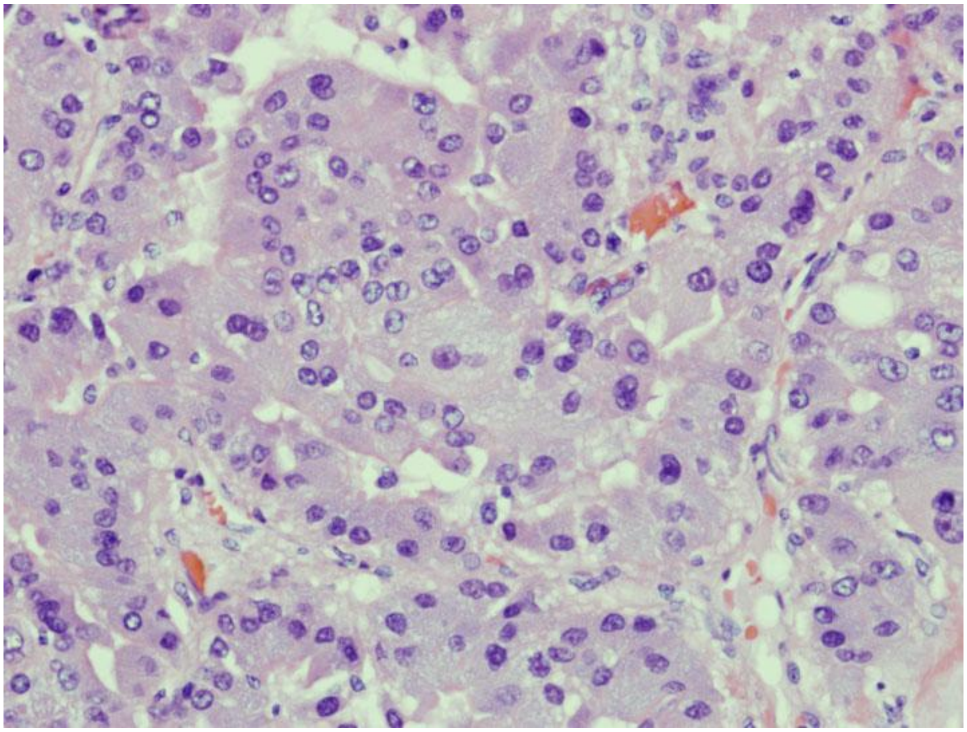

**FIGURE 5.** Tumor cells have abundant granular cytoplasm, round nuclei, and prominent nucleoli, features characteristic of hepatocytes.

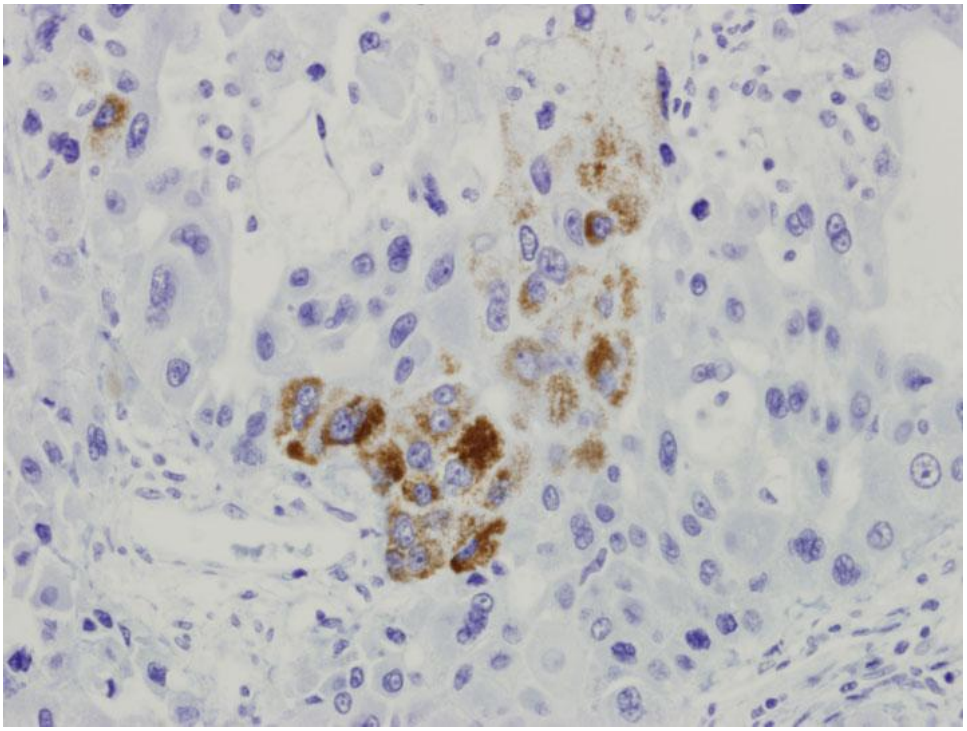

**FIGURE 6.** Hepar immunostain positive in tumor cells, confirming the diagnosis of HCC.

## COMMENT

Large renal masses are most often RCC, but the differential diagnosis includes oncocytoma, angiomyolipoma, urothelial carcinoma, sarcoma, or primary adrenal tumor. Invasion of primary RCC into adjacent structures other than the ipsilateral adrenal is rare without evidence of systemic disease, because RCC typically grows locally with displacement of adjacent structures due to retroperitoneal location and the natural protective barrier of Gerota's fascia[1]. Pathologic involvement of adjacent organs by RCC cannot be reliably predicted from radiographic findings[1]. Urothelial carcinoma typically remains localized in the renal pelvis or locally spreads to lymph nodes, while sarcoma and adrenal carcinoma frequently invade adjacent structures.

Metastatic spread to the kidney is relatively common, with 7.2% of autopsied cancer patients having renal metastasis[2]. The most common manifestation of secondary spread of metastatic disease to the kidneys is bilateral spread in the context of widely disseminated disease. In these circumstances, the most common primary sites of origin are lung (20%), breast (12%), and stomach (11%)[3].

Secondary renal metastases from HCC are uncommon, although there have been cases cited in the literature[4]. One such case described by Aron et al. involved a 74-year-old male with a large mass replacing most of the left lobe of the liver, and a second mass lesion in the postero-inferior part of the right kidney. Both lesions were found to be HCC on pathologic examination[4]. In the context of HCC, extrahepatic spread is found in approximately 11.2% of cases, with the site of first metastasis being lung (42%), bone (24.4%), lymph node (21%), and adrenal gland (9.1%)[6]. Extrahepatic direct extension of HCC is rare at the time of initial diagnosis, while hematogenous metastasis is more common[6].

The adrenal gland is a common site of HCC metastasis. Ohwada et al. reported a case of a 60-year-old man with a large adrenal tumor with a small intrahepatic tumor that showed HCC after surgical resection[7]. Nonencapsulated regions of the liver opening to superior aspect of perirenal space permits direct extension of liver masses from this region[7]. Cases of HCC metastasis to adrenal glands, although rare in clinical practice, are reported in the literature and these cases are often in the context of known hepatitis, previously treated HCC, or in patients with known cirrhotic lesions[8].

Our patient was found to have primary HCC with direct extension into the kidney, which is extremely uncommon. Hsu et al. reported a case of a 50-year-old male with a right kidney tumor in contact with a tumor in the right lobe of the liver, with evidence of invasion into the capsule of the hepatic mass, and both tumors were histopathologically determined to be HCC[5]. If the tumor in our case was suspected to be HCC, the diagnostic approach would have incorporated measures of serum AFP, hepatitis serologies, and possibly a liver biopsy prior to surgery. Surgical resection as was done would still likely have been the treatment of choice in this case regardless of tumor histology due to its size, location, and favorable degree of residual noncirrhotic liver. Alternative primary treatment modalities of HCC include preoperative or definitive transarterial chemoembolization, radiofrequency ablation, liver transplantation, and molecular therapies[10]. Due to this lesion’s large size, ablative techniques or liver transplant would not be utilized. Interferon alpha can be used as an adjunct after resection of HCC secondary to viral hepatitis, with randomized studies reporting improved overall survival[11,12]. The average 5-year survival for stage IV HCC that is aggressively treated is 45%[13].

The tumor in the current case was suspected to be adrenal vs. renal in origin based on radiographic findings, but postoperatively was pathologically diagnosed as primary HCC. Preoperative tissue biopsy can be considered to help identify the primary tumor type, although biopsy is not typically obtained in cases of RCC or HCC. In our patient, without a known history of liver disease, HCC was not apparent. Mild LFT elevation was attributed to direct extension of the mass. Clues to the diagnosis of primary HCC include a history of chronic hepatitis or cirrhosis. The patient in this case originated from Ghana, which is a region endemic for hepatitis, and painful large hepatic masses are a common presentation of HCC in Africa[9].

## CONCLUSION

When approaching a large renal mass with suspected direct invasion of the liver, the possibility of primary HCC should be considered. Primary HCC with direct renal extension is an exceedingly rare occurrence, but the tumor marker AFP and clinical risk factors (hepatitis, cirrhosis) may improve on the limitations of radiographic imaging.
